# Defibrotide Prophylaxis of Sinusoidal Obstruction Syndrome in Adults Treated With Inotuzumab Ozogamicin Prior to Hematopoietic Stem Cell Transplantation

**DOI:** 10.3389/fonc.2022.933317

**Published:** 2022-06-15

**Authors:** Fabio Giglio, Elisabetta Xue, Raffaella Greco, Lorenzo Lazzari, Daniela Teresa Clerici, Francesca Lorentino, Sara Mastaglio, Sarah Marktel, Maria Teresa Lupo-Stanghellini, Magda Marcatti, Consuelo Corti, Massimo Bernardi, Simona Piemontese, Fabio Ciceri, Jacopo Peccatori

**Affiliations:** ^1^ Haematology and Bone Marrow Transplant Unit, IRCCS San Raffaele Scientific Institute, Milan, Italy; ^2^ PhD Program in Public Health, Department of Medicine and Surgery, University of Milano-Bicocca, Milan, Italy; ^3^ University Vita-Salute San Raffaele, Milan, Italy

**Keywords:** Inotuzumab ozogamicin, defibrotide, prophylaxis, SOS, VOD, Allo-HCT, allogeneic hematopoietic stem cell transplantation

## Abstract

Sinusoidal Obstruction Syndrome (SOS) is a life threatening HSCT complication and it can rapidly evolve in Multiple Organ Dysfunction Syndrome, with a mortality exceeding 80%. Early treatment with defibrotide is the leading factor for efficacy. Its prophylactic use is recommended in the pediatric setting, but its value isn’t validated for adults, although factors for individual risk assessment are debated. We here present a real-world experience of Defibrotide prophylaxis in adults at very high risk of SOS. We treated with prophylactic Defibrotide and Ursodeoxycholic Acid seven patients receiving allogeneic HSCT for high risk B-ALL, previously treated with single agent Inotuzomab-Ozogamicin. They all had other high risk factors for SOS such as previous hepatotoxicity, previous allo-HSCT, double alkylating conditioning. All patients received Treosulfan-Fludarabine conditioning, Thiotepa was added in 4 patients and 4GyTBI in 2 patients. GvHD prophylaxis included post-transplant cyclophosphamide, rapamycin and mycophenolate. Donor source was PBSC. Five patients received family MMRD transplant, 1 patient a MRD transplant and 1 patient a MUD transplant. Non-severe gastrointestinal bleeding occurred in two patients requiring defibrotide temporarily discontinuation. SOS occurred in 3/7 cases within 21 days after HSCT and no late-onset SOS were diagnosed. SOS caused death in all cases. All three patients were characterized by a common pattern of very high risk factors by prior HSCT, they all received a myeloablative conditioning with Treosulfan-Thiotepa and a MMRD transplant. Defibrotide prophylaxis apparently failed to protect against the development of SOS in those patients treated with a double alkylator-based conditioning regimen, while a possible efficacy for the other high-risk patients is debatable.

## Introduction

Hepatic Sinusoidal Obstruction Syndrome (SOS) is a potentially life-threatening complication of Hematopoietic Stem Cell Transplantation (HSCT). SOS pathogenesis is related to pro-inflammatory signals that determine the activation of hepatic stellate cells with subsequent collagen deposit, progressive sinusoidal obstruction, and portal hypertension. While mild SOS are underdiagnosed and spontaneously resolve, severe cases might evolve in liver dysfunction and multiple organ failure (MOF). Given the lack of pathognomonic features, several groups tried to identify clinical and laboratory criteria that define SOS (1). Several transplant, patient, and liver-related risk factors have been suggested (2) and, among them, the administration of Inotuzumab ozogamicin (IO), an anti-CD22 calicheamicin-linked monoclonal antibody approved for B-cell acute lymphoblastic leukemia (B-ALL), has been recognized as a major risk factor for both drug-induced liver injury and SOS (3). Given the lack of CD22 expression on liver cells, it has been hypothesized that drug-induced hepatic damage is secondary to the disposition of the calicheamicin metabolites by hepatocytes and the subsequent biliary excretion, which might expose liver cells to toxic injury; moreover calicheamicin uptake by endothelial cells might determine cell toxicity associated with platelet sequestration in liver sinusoids causing their obstruction (4, 5). Anti-inflammatory and anti-thrombotic properties of defibrotide, a mixture of oligonucleotides purified from porcine gut mucosa, reduce endothelial damage and potentially revert the cascade of organ dysfunction; therefore, defibrotide has been approved for severe SOS treatment (6). Whereas multiple dosages of defibrotide have been tested (7), the actual recommendations for the treatment of SOS indicate 25 mg/kg/day, administered for a minimum of 21 days, as the standard dose. Regarding prophylactic therapies, there is only low-quality evidence on the efficacy of defibrotide and ursodeoxycholic acid (UDCA) in reducing the incidence of SOS (8–10). The seminal paper by Corbacioglu et al. showed a borderline benefit in SOS incidence in children receiving prophylactic defibrotide that did not translate in overall survival improvement (10), and no clear data support its use in adults. Furthermore, the role of defibrotide in patients previously treated with IO is unknown. Herein, we present a real-world experience of peri-HSCT defibrotide prophylaxis in adult patients at risk of SOS because of a history of IO administration.

## Methods and Results

From May 2016 to April 2018, seven patients diagnosed with relapsed/refractory B-ALL with a history of IO administration underwent peripheral blood allogeneic HSCT at our Institution. Patient and transplant characteristics are shown in **Table 1**. IO was given as single agent for either one (n=1) or two cycles (n=6) at standard dose, with the last dose being administered at a median time of 41.5 days (range 34-61) prior to HSCT. Conditioning regimens were based on treosulfan (42 g/m^2^) and fludarabine (150 mg/m^2^) in all cases; thiotepa was added in four patients, whereas two cases received also 4Gy total body irradiation. As per institutional policy, graft versus host disease (GvHD) prophylaxis consisted in post-transplant cyclophosphamide (PT-Cy) on day +3 and day +4, rapamycin and mycophenolate. All patients received peri-transplant UDCA 300 mg twice daily. Defibrotide was administered intravenously in four daily doses for a total dose of 25 mg/kg/day, starting the first day of conditioning and throughout engraftment. Informed consent for off-label use of prophylactic defibrotide was signed by all patients.

**Table 1:** Patient, disease, and treatment characteristics.

Patient	#1	#2	#3	#4	#5	#6	#7
Sex and age	M, 25 y	M, 36 y	F, 22 y	M, 30 y	M, 21 y	M, 38 y	M, 26 y
Disease - status	B-ALL, active disease	B-ALL active disease	B-ALL, active disease	B-ALL, complete remission	B-ALL, complete remission	B-ALL, complete remission	B-ALL, complete remission
Previous lines of therapy	8	4	6	4	5	2	6
Previous allo-HSCT	yes	no	yes	yes	yes	yes	no
Pre-existing liver abnormalities	G2* Bilirubin and ALT increase	G2* ALT increase; mild steatosis	no	no	no	no	no
IO cycles numbers (cumulative dose)	2 cycles (3.3 mg/mq)	2 cycles (3.3 mg/mq)	2 cycles (3.3 mg/mq)	2 cycles (3.3 mg/mq)	2 cycles (3.3 mg/mq)	2 cycles (3.3 mg/mq)	1 cycle (1.8 mg/mq)
Days from IO to HSCT	25	61	49	25	34	60	49
Conditioning	Treo/Flu TBI4Gy	Treo/Flu TBI4Gy	Thio/Treo/Flu	Thio/Treo/Flu	Thio/Treo/Flu	Thio/Treo/Flu	Treo/Flu
Type of transplant	Mismatched related	Matched related	Mismatched related	Mismatched related	Mismatched related	Mismatched related	Matched unrelated
Days to engraftment	29	30	Died in aplasia	19	15	18	35
Days of defibrotide	27	32	11	35	33	35	64
**SOS diagnosis**	no	no	no	**YES**	**YES**	**YES**	no
Days from HSCT to SOS	na	na	na	10	13	9	na
Days of follow up	116	1092	11	28	26	28	229
Cause of death	Disease relapse	Disease relapse	Septic shock	SOS	SOS	SOS	Disease relapse.

B-ALL B-cell acute lymphoblastic leukemia; HSCT allogeneic Hematopoietic Stem Cell Transplantation; IO Inotuzumab ozogamicin; Treo/Flu Treosulfan/Fludarabine; Thio/Treo/Flu Thiotepa/Treosulfan/Fludarabine; SOS sinusoidal obstruction syndrome.

*According to CTCAE 5.0. bold values means to better identify case with SOS..

na, not applicable.

The median duration of defibrotide administration was 33 days (range, 11-64); the drug was overall well tolerated as it was temporarily discontinued only in two cases, respectively for two and four days, due to transient gastrointestinal bleeding (CTCAE v5.0 grade 3); no other adverse reactions were documented. Six patients engrafted after 18 days (range, 15-35), whereas one deceased in aplasia. One patient developed acute GvHD and required multiple lines of immunosuppressants; we documented one case of post-transplant microangiopathy shortly after SOS diagnosis.

Three out of seven patients (#4, #5, #6) developed all classical signs of SOS (hyperbilirubinemia, ascites, painful hepatomegaly, and weight gain) (1) a few days before engraftment, with a rapid evolution into a very severe form (2). They all received a double alkylator chemoconditioning; patient #3 also received the same chemoconditioning but died in aplasia at +11 post HSCT. No diagnostic liver biopsy was performed given the high risk of procedural hemorrhagic complications. SOS determined MOF and death in all three cases.

## Discussion

We here describe a real-world case series of patients at very high risk of SOS and 3 out of 7 patients developed this severe complication. These three patients showed common features: all were young adult males transplanted from a haploidentical donor for a B-ALL in complete remission after 2 cycles of IO; moreover, all had a history of a previous allogeneic HSCT and received a myeloablative conditioning with double alkylating agents prior to HSCT.

It is noteworthy that all patients in our series, including those not diagnosed with this complication, displayed at least one additional risk factor of SOS such as pre-existing hepatic abnormalities, active disease, rapamycin use, conditioning with TBI, use of HLA-mismatched donors, and previous allogeneic HSCT (1, 2, 6). Double alkylators and pre-HSCT hepatic abnormalities have been specifically associated with SOS in patients pre-treated with IO (3) and these risk factors were present in two and four patients, respectively (**Table 1**).

All these factors were scattered among patients whit and without SOS and, in this very small cohort, it is not possible to discriminate the role of each risk factor. However, it appears evident that those who were treated with double alkylator conditioning developed SOS despite prophylaxis with defibrotide plus UDCA; the other patient who also received thiotepa inside the conditioning died at day +11 after HSCT, preventing us from assessing the effectiveness of prophylaxis (**Table 1**). It is also worth mentioning the use of PT-Cy as GVHD prophylaxis in this case series, so that the three patients developing SOS effectively received three alkylators. It is not clear at this point if PT-Cy should be regarded as a risk factor for SOS, but it surely warrants further investigations.

All diagnoses occurred right before engraftment and the clinical picture evolved dramatically within few days, making SOS diagnosis striking. No differences in the number of IO cycles (never more than 2) and interval from last IO to HSCT were evident between patients diagnosed with SOS and those who did not develop this complication.

Whereas the approval of IO has greatly improved the treatment armamentarium for relapsed/refractory B-ALL, emergence of hepatic toxicity, namely SOS, has questioned the safety of this new drug. Recently, a learning curve has allowed a better use of IO, specifically as a bridge to allogeneic HSCT (11). In the setting of prophylaxis, Corbacioglu and colleagues reported a decrease of SOS incidence from 20% to 12% in a phase 3 trial conducted in a high-risk pediatric population, including patients who received gentuzumab ozogamicin but not IO (10). On the contrary, the role of prophylaxis is particularly debated in adult patients, where no evidence-based data were able to demonstrate its efficacy (9). Our experience suggests that defibrotide prophylaxis is safe in the adult population, with only two documented bleeding events promptly resolved with supportive care and temporary drug interruption. However, the drug, in association with UDCA, was only partially effective in preventing SOS. Our data confirm the detrimental effect of double alkylator conditioning for patients undergoing HSCT previously treated with IO, a feature that we now recognize as one of the most important risk factors for SOS in this treatment setting (3, 11). Furthermore, recent data confirmed the benefit of a TBI-based conditioning in children with ALL compared to double alkylator chemoconditioning in terms of both relapse and treatment related mortality (12). This approach should be considered as reasonable also in young adults and warrants investigations in the setting of IO pre-treated patients at risk for SOS.

Indeed, in our study, defibrotide prophylaxis apparently failed to protect against the development of SOS only in those patients treated with a double alkylator-based conditioning regimen, while a possible efficacy could be at least presumed for the other high-risk patients.

In conclusion, with the caution derived from analyzing the results of a small single-center case series, the use of peri-HSCT defibrotide prophylaxis in adult patients at very high-risk of SOS due to a previous history of IO exposure demonstrated to be safe and partially effective. Minimization of other known cumulative risk factors should be pursued to further reduce the incidence of SOS in this population, and, particularly, the addition of a second alkylating agent into the conditioning scheme must be avoided. Other tips, such as further reducing the number of IO administrations, longitudinal monitoring with liver ultrasound elastography to allow early diagnosis and avoiding other hepatotoxic drugs should be considered (13). Only well-designed prospective clinical trials could demonstrate the usefulness of defibrotide in this setting.

## Data Availability Statement

The raw data supporting the conclusions of this article will be made available by the authors, without undue reservation.

## Ethics Statement

This study was reviewed and approved by San Raffaele Institutional Ethical Committee. Written informed consent was obtained from all participants for their participation in this study.

## Author Contributions

FG and FC designed the study. FG and EX wrote the manuscript. All authors were involved in patients management. All authors interpreted results and validated the manuscript’s content. All authors contributed to the article and approved the submitted version.

## Conflict of Interest

The authors declare that the research was conducted in the absence of any commercial or financial relationships that could be construed as a potential conflict of interest.

## Publisher’s Note

All claims expressed in this article are solely those of the authors and do not necessarily represent those of their affiliated organizations, or those of the publisher, the editors and the reviewers. Any product that may be evaluated in this article, or claim that may be made by its manufacturer, is not guaranteed or endorsed by the publisher.

## References

[1] Bonifazi F Sica S Angeletti A Marktel S Prete A Iori AP. Veno-Occlusive Disease in HSCT Patients: Consensus-Based Recommendations for Risk Assessment, Diagnosis, and Management by the GITMO Group. Transplant (2021) 105(4):686–94. doi: 10.1097/TP.0000000000003569 33273315

[2] Mohty M Malard F Abecassis M Aerts E Alaskar AS Aljurf M. Revised Diagnosis and Severity Criteria for Sinusoidal Obstruction Syndrome/Veno-Occlusive Disease in Adult Patients: A New Classification From the European Society for Blood and Marrow Transplantation. Bone Marrow Transpl (2016) 51(7):906–12. doi: 10.1038/bmt.2016.130 PMC4935979 27183098

[3] Kantarjian HM DeAngelo DJ Advani AS Stelljes M Kebriaei P Cassaday RD. Hepatic Adverse Event Profile of Inotuzumab Ozogamicin in Adult Patients With Relapsed or Refractory Acute Lymphoblastic Leukaemia: Results From the Open-Label, Randomised, Phase 3 INO-VATE Study. Lancet Haematol (2017) 4(8):e387–e98. doi: 10.1016/S2352-3026(17)30103-5 28687420

[4] McDonald GB Freston JW Boyer JL DeLeve LD. Liver Complications Following Treatment of Hematologic Malignancy With Anti-CD22-Calicheamicin (Inotuzumab Ozogamicin). Hepatol (2019) 69(2):831–44. doi: 10.1002/hep.30222 PMC6351187 30120894

[5] Guffroy M Falahatpisheh H Biddle K Kreeger J Obert L Walters K. Liver Microvascular Injury and Thrombocytopenia of Antibody-Calicheamicin Conjugates in Cynomolgus Monkeys-Mechanism and Monitoring. Clin Cancer Res (2017) 23(7):1760–70. doi: 10.1158/1078-0432.CCR-16-0939 27683177

[6] Mohty M Battista ML Blaise D Calore E Cesaro S Maximova N. A Multicentre, Multinational, Prospective, Observational Registry Study of Defibrotide in Patients Diagnosed With Veno-Occlusive Disease/Sinusoidal Obstruction Syndrome After Haematopoietic Cell Transplantation: An EBMT Study. Bone Marrow Transpl (2021) 56(10):2454–63. doi: 10.1038/s41409-021-01265-2 34059801

[7] Corbacioglu S Carreras E Mohty M Pagliuca A Boelens JJ Damaj G. Defibrotide for the Treatment of Hepatic Veno-Occlusive Disease: Final Results From the International Compassionate-Use Program. Biol Blood Marrow Transpl (2016) 22(10):1874–82. doi: 10.1016/j.bbmt.2016.07.001 27397724

[8] Dignan FL Wynn RF Hadzic N Karani J Quaglia A Pagliuca A. BCSH/BSBMT Guideline: Diagnosis and Management of Veno-Occlusive Disease (Sinusoidal Obstruction Syndrome) Following Haematopoietic Stem Cell Transplantation. Br J Haematol (2013) 163(4):444–57. doi: 10.1111/bjh.12558 24102514

[9] Cheuk DK Chiang AK Ha SY Chan GC. Interventions for Prophylaxis of Hepatic Veno-Occlusive Disease in People Undergoing Haematopoietic Stem Cell Transplantation. Cochrane Database Syst Rev (2015) 5):CD009311. doi: 10.1002/14651858.CD009311.pub2 PMC10891422 26017019

[10] Corbacioglu S Cesaro S Faraci M Valteau-Couanet D Gruhn B Rovelli A. Defibrotide for Prophylaxis of Hepatic Veno-Occlusive Disease in Paediatric Haemopoietic Stem-Cell Transplantation: An Open-Label, Phase 3, Randomised Controlled Trial. Lancet (2012) 379(9823):1301–9. doi: 10.1016/S0140-6736(11)61938-7 22364685

[11] Kebriaei P Cutler C de Lima M Giralt S Lee SJ Marks D. Management of Important Adverse Events Associated With Inotuzumab Ozogamicin: Expert Panel Review. Bone Marrow Transpl (2018) 53(4):449–56. doi: 10.1038/s41409-017-0019-y PMC5897380 29330398

[12] Peters C Dalle JH Locatelli F Poetschger U Sedlacek P Buechner J. Total Body Irradiation or Chemotherapy Conditioning in Childhood ALL: A Multinational, Randomized, Noninferiority Phase III Study. J Clin Oncol (2021) 39(4):295–307. doi: 10.1200/JCO.20.02529 33332189 PMC8078415

[13] Marks DI Kebriaei P Stelljes M Gökbuget N Kantarjian H Advani AS. Outcomes of Allogeneic Stem Cell Transplantation After Inotuzumab Ozogamicin Treatment for Relapsed or Refractory Acute Lymphoblastic Leukemia. Biol Blood Marrow Transpl (2019) 25(9):1720–9. doi: 10.1016/j.bbmt.2019.04.020 31039409

